# The ER Aminopeptidases, ERAP1 and ERAP2, synergize to self-modulate their respective activities

**DOI:** 10.3389/fimmu.2022.1066483

**Published:** 2022-12-08

**Authors:** Adrian Martín-Esteban, Jesus Contreras Rodriguez, David Peske, Jose A. Lopez de Castro, Nilabh Shastri, Scheherazade Sadegh-Nasseri

**Affiliations:** ^1^ Department of Pathology, Immunopathology Division, Johns Hopkins University, Baltimore, MD, United States; ^2^ Centro de Biología Molecular Severo Ochoa (CSIC-UAM), Madrid, Spain

**Keywords:** HLA, antigen processing, aminopeptidase, peptidome, mechanism, epitope generation

## Abstract

**Introduction:**

Critical steps in Major Histocompatibility Complex Class I (MHC-I) antigen presentation occur in the endoplasmic reticulum (ER). In general, peptides that enter the ER are longer than the optimal length for MHC-I binding. The final trimming of MHC-I epitopes is performed by two related aminopeptidases, ERAP1 and ERAP2 in humans that possess unique and complementary substrate trimming specificities. While ERAP1 efficiently trims peptides longer than 9 residues, ERAP2 preferentially trims peptides shorter than 9 residues.

**Materials and Methods:**

Using a combination of biochemical and proteomic studies followed by biological verification.

**Results:**

We demonstrate that the optimal ligands for either enzyme act as inhibitors of the other enzyme. Specifically, the presence of octamers reduced the trimming of long peptides by ERAP1, while peptides longer than nonomers inhibit ERAP2 activity.

**Discussion:**

We propose a mechanism for how ERAP1 and ERAP2 synergize to modulate their respective activities and shape the MHC-I peptidome by generating optimal peptides for presentation.

## Introduction

Cytotoxic CD8+ T-cells survey for pathogenic perturbations in the peptidome by interacting with the surface MHC-I molecules in complex with peptide fragments derived from intracellular proteins. Endoplasmic Reticulum Aminopeptidase associated with Antigen Processing (ERAAP), was originally discovered as an enzyme critical for the final trimming of the peptides loaded onto MHC-I (1). In mice, loss of ERAAP results in decreased surface MHC-I, and an altered peptidome including the prevalence of alternative long peptides (2, 3).

In humans, two homologous enzymes, ERAP1 and ERAP2 can act in concert to perform the analogous function of murine ERAAP (4). The importance of ERAPs to human disease has been extensively documented (5). Several autoimmune diseases such as ankylosing spondylitis, birdshot chorioretinopathy, beçhet disease and psoriasis –in combination with population-specific HLAs– are linked to ERAP1 and ERAP2 function. Alterations in the peptide repertoire presented by HLA resulting from the enzymatic activity of ERAP1 haplotypes and differential expression of ERAP2 underlie the pathology associated with these diseases (6–11). Although these two aminopeptidases play similar roles in antigen presentation, they possess diametrically different enzymatic activities as well as differences in substrate specificities (12–15).

Despite sharing ~50% homology, ERAP1 and ERAP2 have distinct enzymatic activities and substrate preferences (5). ERAP1 has been proposed to rely on a molecular ruler mechanism to optimally trim peptides longer than 9 residues (13) (16). In contrast, ERAP2 most efficiently trims peptides shorter than 10 residues (15). ERAP1 and ERAP2 are well characterized in isolation but how the two enzymes and their products affect each other remains unclear. ERAP1 and ERAP2 have individually been crystalized with inhibitors and peptide analogues- the two enzymes, however, have not yet been co-crystalized. However, molecular dynamics studies predict the possibility of a heterodimer formation that might be more efficient in enzymatic activities, but evidence to document their activities in living cells remains to be described (17–19).

Due to the complexities involved, an understanding of the dynamics of the interactions among all of the substrates, products, and enzymes during ERAP1/ERAP2 mediated peptide trimming has been challenging. Here, we employed various biochemical and enzymological strategies in conjunction with peptidomic approaches to elucidate interactions unique to the ERAP1 and ERAP2 aminopeptidases. Our results reveal a model unifying the enzymatic activities of ERAP1 and ERAP2 dependent on cross-feedback between the enzymes and the peptide pools generated by their respective activities. Understanding the synergy between these related aminopeptidases is crucial for uncovering alternative pharmaceutical targets to harness and modulate immune factors while minimizing the risk of system-wide pathologies in the treatment of cancers and autoimmune conditions.

## Material and methods

### Generation of recombinant baculovirus

cDNAs encoding ERAP1 Hap 2 (K528, most active variant) and ERAP2 were confirmed by Sanger sequencing, cloned into the pFastBac vector and used to transform competent DH10Bac *E. coli* (Gibco) Recombinant bacmids were isolated by standard DNA preparation methods and used to transfect Hi-5 adherent insect cells with Cellfectin II (Invitrogen) to produce recombinant baculoviruses. Supernatant from transfected cells was isolated after 72h, and the titer was determined by immunofluorescence plaque assay with mouse anti-6His (Qiagen, Hilden, Germany) and Alexa 488 donkey anti-mouse antibodies (Invitrogen), at 1:400 and 1:500 dilutions, respectively. Concentrated virus stocks were produced by infecting Hi-5 adherent cell cultures at a high multiplicity of infection and collecting the supernatant after 72h.

### Protein expression and purification

Recombinant ERAP1 proteins were produced in nonadherent Hi-5 insect cells grown in Express Five serum-free medium (Invitrogen). Following infection with baculovirus carrying the ERAP1 or ERAP2 constructs, the culture medium containing secreted enzymes was harvested by centrifugation (3000xg, 30min at 4°C). The supernatant was concentrated in a Stirred Ultrafiltration Cell (Amicon, Millipore), adjusted to 50 mM phosphate, 300 mM NaCl, 10 mM imidazole pH 8.0, and loaded onto a Poly-prep Chromatography Column (Bio-Rad) pre-loaded with nickel-nitrilotriacetic acid agarose (Qiagen). The column was washed with a buffer of the same composition as above adjusted to 20 mM imidazole, elution was performed with a 40 –150 mM imidazole gradient. Protein elution was confirmed *via* SDS-PAGE. Protein-containing fractions were dialyzed in Vivaspin 500 (Sartorius Stedim Biotech, Goettingen, Germany) MWCO columns against 50 mM Tris/HCl buffer, 1 mM DTT, pH 7.4, aliquoted, and stored at -70°C.

### Fluorogenic substrate hydrolysis assay

3μg Arg-7-amido-4-methylcoumarin (R-AMC) (Bachem Distribution, Wei am Rhein, Germany) in 50μl of 1 M Tris/HCl buffer, pH 8.0, was mixed with 100 ng of ERAP2 in an equal volume of 50 mM Tris/HCl, 1 mM DTT, pH 7.4 (E/S ratio,1:30), and incubated at 37°C up to 1 h. Substrate hydrolysis was assessed by measuring the fluorescence of free AMC at 14-s intervals, at 380nm and 460 nm excitation and emission wavelengths, respectively, in a Fluostar Optima Multiwave Plate Reader (BMG Labtec, Ortenberg, Germany).

### Peptide trimming

Synthetic peptides (purity >90%) were purchased from GenScript. Two nanograms per microliter of each ERAP1 and ERAP2 were incubated with 20 ng/μl of each peptide (enzyme: substrate ratio 1:10) in 50 mM Tris HCl buffer, 1 mM dithiothreitol, pH 7.4, at 37°C in a total volume of 10 μl per time point. Aliquots of 10 μl of the digestion mixture were removed at various times, and the reaction was stopped with 1.5 μl of 5% trifluoroacetic acid. The samples were purified with OMIX C18 pipette tips (Varian) and analyzed by MALDI-TOF MS in positive ion reflector mode at 25kV in the mass-to-charge (m/z) range of 400-2200. Peptides yield were estimated on the basis of the relative intensity of the respective ion peaks. This method was validated by comparison with HPLC-based measurements in (20).

## Results

### Selective inhibition of ERAP1 trimming activity by Octamers

**Figure 1:**
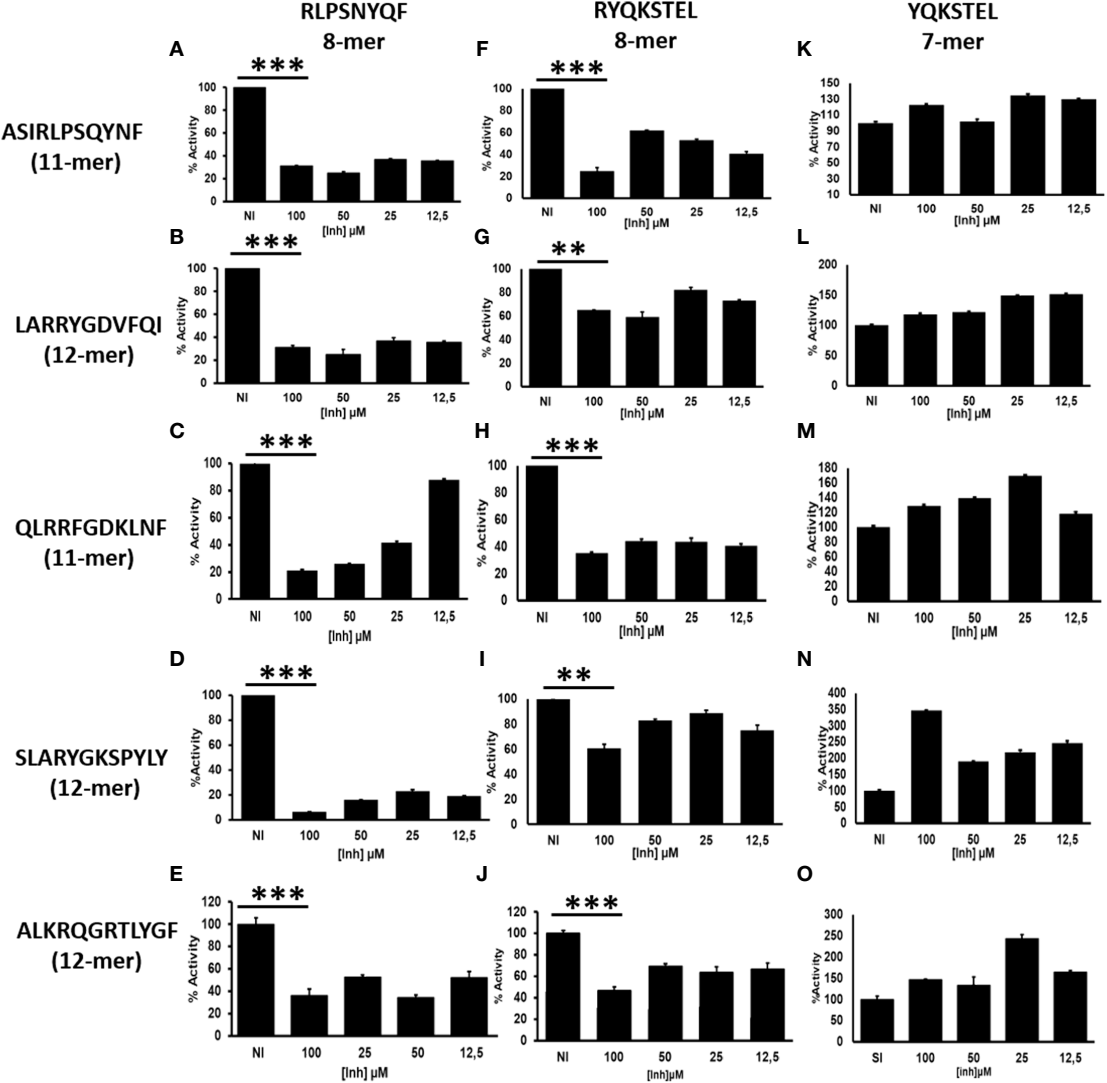
ERAP1 activity is inhibited by octamers, not by heptamer: **(A)**, ERAP1 was incubated with the indicated N-terminally extended peptides (left) and increasing concentration of octamer peptide inhibitor (Vertical; RLPSQYNF panel **(A**–**E)** or RYQKSTEL panel **F–J**), NI (No Inhibitor). **(B)**, The N-terminally extended peptides were incubated with ERAP1 in the presence of increasing concentration of heptamer (YQKSTEL) panel **(K**–**O)**. Trimming reactions were performed for 32 minutes at an E: S ratio of 1:10 and percent enzyme activity was calculated relative to the quantity of fully trimmed product in the NI reaction. Average and SD for each reaction were derived from three experiment. t-test analysis provided statistical significance for mean comparisons (P < 0.05). *** extremely significant, ** very significant and * significant.

Cytosolic peptides ranging from 8-16aa long are preferentially translocated into the ER by TAP (21). The N-terminal amino acids of peptides are trimmed by ERAP1 until they reach an optimal length of 8-10aa for loading onto MHC-I (22, 23). Peptides destined for presentation by MHC-I can also be over-trimmed by ERAP1 leading to the destruction of the epitope and elimination from the peptidome. Octamer epitopes or over trimming products are known to be highly resistant to ERAP1 activity (**Supplementary Figure S1**), but not for ERAP2 (**Supplementary Figure S2A**) (13). However, octamer peptides can activate ERAP1 against a L-AMC substrate (16). Although whether ERAP1 generated octamers negatively influence ERAP1 activity against long peptides remains unknown. To investigate this, we established a model system using HLA-B27-peptidome derived peptides and their N-terminally extended precursors, and subjected those peptides to ERAP1 digestion in the presence or absence of octamer variants. As shown in **Figures 1A–J**, the presence of octameric peptide products decreased the trimming of all tested N-terminally extended peptide substrates by ERAP1. Nonetheless, the extent of inhibition varied by the octamer used and the sequence of the extended peptide. Inhibition of ERAP1 trimming of the long peptide precursors was stronger for the octamer RLPSNYQF (**Figures 1A–E**) as compared to the octamer RYQKSTEL (**Figures 1F–J**). In some cases, low concentrations of the inhibitory octamers were sufficient to decrease ERAP1 activity (**Figures 1A, B, D, F, H**), whereas others required higher octamer concentrations (**Figures 1G, I**). In contrast to the clear inhibitory effect of octamers, the presence of heptamers (**Figures 1K–O**) showed no measurable inhibition, yet we observed an enhancement of ERAP1 trimming in some cases due to the shorter peptides as shown previously (16).

### ERAP2 activity rescues octamer-dependent ERAP1 inhibition

**Figure 2:**
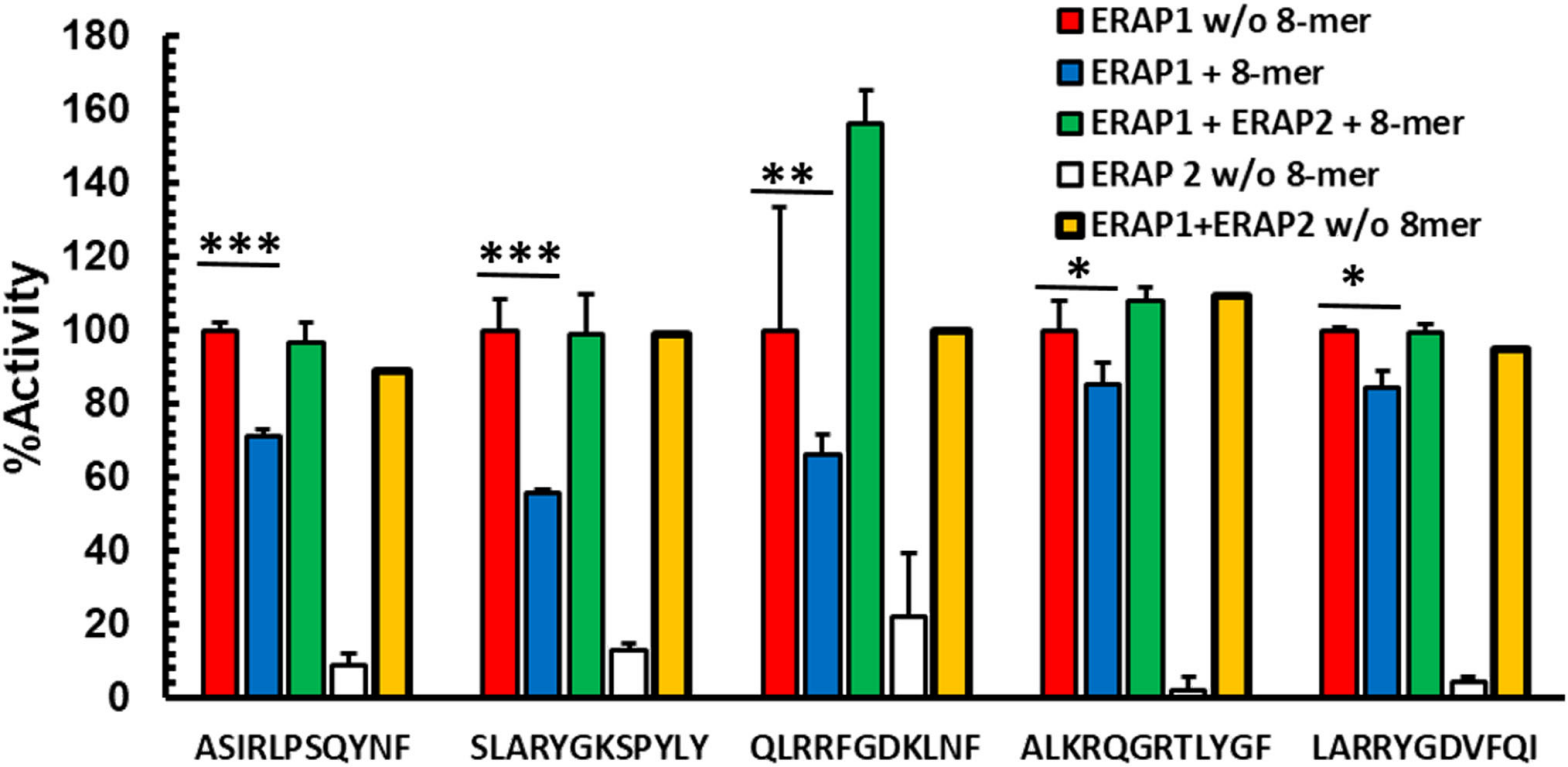
Destruction of octamers by ERAP2 rescues octamer-dependent ERAP1 inhibition Peptides were incubated with ERAP1 at an E: S 1:10 (red), ERAP2 (white) or ERAP1 plus ERAP2 (yellow), in the presence of 100 μM RYQKSTEL (blue) and in presence of inhibitor plus ERAP2 (green) at 32 min. Yields are relative to the total amount of peptide estimated as the added intensity of ion peaks corresponding to each peptide species by MALDI-TOF MS spectrometry of final reaction products. The data are mean +- SD of 3 experiments. t-test analysis provided statistical significance for mean comparisons (P < 0.05). *** extremely significant, ** very significant and * significant.

While ERAP1 has its optimal trimming activity against longer peptides, the preferred substrates for ERAP2 are shorter than 10 amino acids; ERAP2 has its strongest activity against nonamers and octamers (14, 15). We, therefore, hypothesized that ERAP2 mediated destruction of octamers might reduce inhibition of ERAP1 in a mixed enzymatic reaction. To test this hypothesis, we measured the kinetics of ERAP1 trimming of N-terminally extended peptides in the presence of inhibitory octameric peptide with or without ERAP2 (**Figure 2**, and **Supplementary Figure S3**). For all combinations tested, the presence of ERAP2 did not enhance destruction of the long peptides by ERAP1 (**Supplementary Figure S4**). Instead, we observed the recovery of ERAP1 activity in the presence of the octameric peptide. Interestingly, for some substrates (QLRRFGDKLNF and ALKRQGRTLYGF), a measurable enhancement of ERAP1 activity was observed. In the case of QLRRFGDKLNF, the observed enhancement occurred rapidly, whereas ALKRQGRTLYFG showed slower kinetics and a more modest enhancement overall **(** **Supplementary Figure S3** **)**. It has been suggested (12, 13, 16) that some peptides shorter than 8 amino acids can bind to and enhance ERAP1 activity by favoring formation of the ERAP1 closed conformation intermediates conducive to optimal substrate binding. Our experimental model supports those observations and further suggests that the ERAP1 modulating short peptides can be generated through the trimming of peptide octamers to shorter lengths by ERAP2 (**Supplementary Figures S2A**, **S2B**). Altogether, these results suggest that ERAP1 activity is selectively inhibited by octamers, while heptamers either have no effect on enzymatic activity, or enhance ERAP1 trimming of the longer peptide substrates. It is possible that by trimming octamers and generating heptamers and shorter peptides, ERAP2 could have a positive influence on the activity of ERAP1.

### Long peptides inhibit ERAP2 activity against a fluorogenic substrate

**Figure 3:**
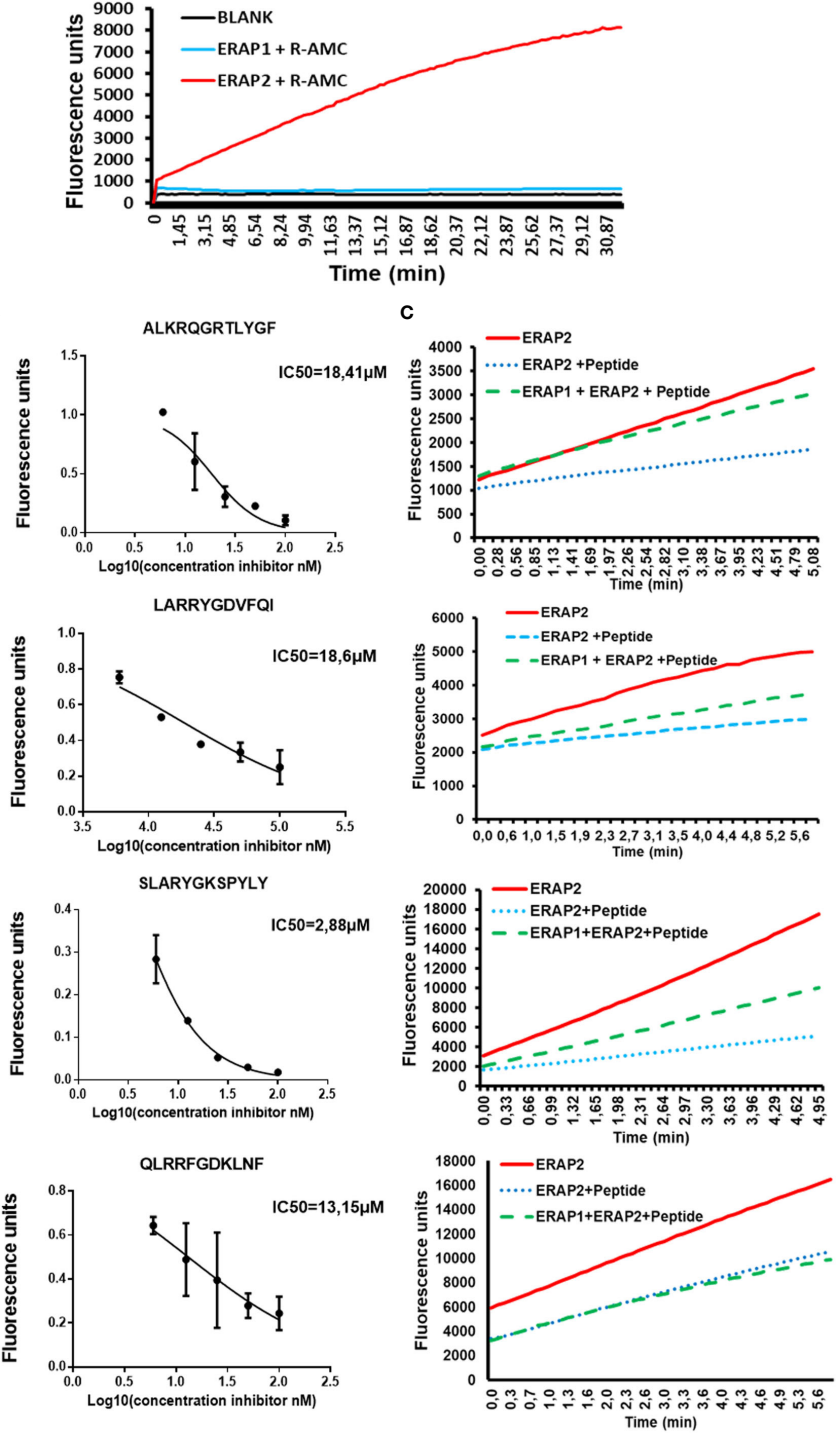
ERAP1 activity can rescue inhibition of ERAP2 R-AMC hydrolysis by long peptides **(A)**; R-AMC hydrolysis time course [E:S, 1:30) (w/w), ERAP2 alone (Black), ERAP2 and R-AMC (Red), ERAP1 with R-AMC (Blue). AMC fluorescence was measured as a function of time. **(B)**] Increasing Long peptide concentrations were incubated with ERAP2 and R-AMC, IC50 for each peptide was calculated. **(C)**; ERAP2 was incubated with R-AMC in the absence of peptide (Solid Red), or the IC50 for each long peptide (Dotted Blue). Long peptides were pre-incubated with ERAP1 for 32min, products were purified and added to a reaction containing ERAP2 and R-AMC (Dashed Green). The data are the mean of 3 experiments.

Next, we investigated if peptide length might also influence ERAP2 activity. Because of the difficulties in detecting shorter peptides generated by ERAP2 trimming, we utilized a sensitive Fluorogenic AMC substrate as a function of time per a previous study (15). We tested the ability of ERAP2 to hydrolyze R-AMC (Arginine-AMC) to form the fluorogenic substrate AMC in the presence of the long peptides. First, we determined that R-AMC is trimmed exclusively by ERAP2 and not by ERAP1 (**Figure 3A**), and next, we measured the IC_50_ of each long peptide in the presence of the R-AMC reporter system (**Figure 3B**). Using the determined concentration for each peptide, we measured the trimming kinetics of R-AMC. In all cases, presence of the longer peptides reduced the hydrolysis of the AMC substrate as a function of time. Interestingly, as with ERAP1 inhibition by octamers, the level of inhibition varied according to the nature of the peptides. We then probed the influence of ERAP1 activity on the inhibition of ERAP2 by long peptide substrates (**Figure 3C**). Strikingly, when preincubated with ERAP1, we observed different effects on the activity of ERAP2 dependent on the corresponding P1 residue of the peptide substrate **(** **Supplementary Figure S5**) and the internal sequence of the peptides as reported (24). In the case of the peptide ALKRQGRTLYGF, ERAP2 activity was completely restored, whereas peptides-SLARYGKSPYLY and LARRYGDVFQI only partially restored ERAP2 activity. However, the peptide-QLRRFGDKLNF did not alter ERAP2 inhibition measurably. Thus, our ERAP1 digestion of the peptide inhibitors had different effects on the recovery of ERAP2 activity.

### Digestion of Long Peptides by ERAP1 Leads to recovery of ERAP2 Activity

**Figure 4:**
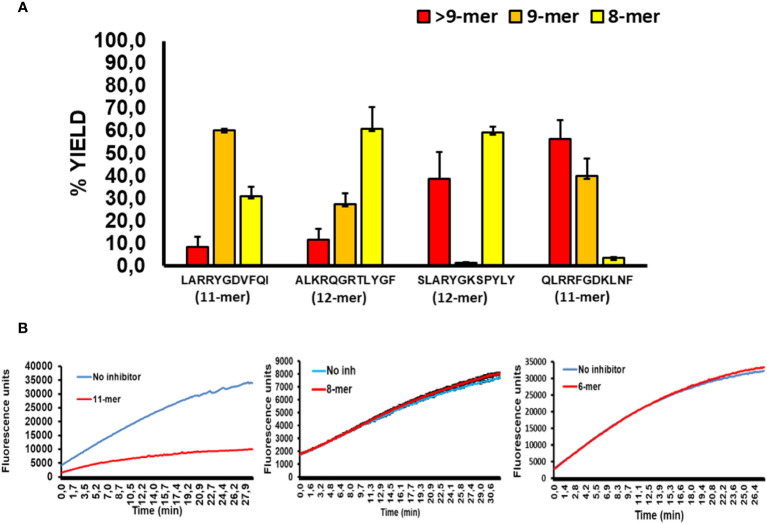
ERAP2 activity is unaffected by octamer and shorter peptides, and ERAP1 long peptide digestion products **(A)**; ERAP1 reaction products from previous long peptide substrates, digestions were performed for 32min. Recovery of long peptide digestion products longer than 9 amino acids (Red), 9-mers (Orange), or 8-mers (Yellow). The data are the mean of 3 experiments. **(B)**; Time course of ERAP2 activity against R-AMC in presence or absence of an 11-mer (EIRRYQKSTEL), 8-mer(RYQKSTEL) and the 6-mer (QKSTEL).

To better evaluate the relationship between the sequence of long peptide inhibitors of ERAP2 and their trimming by ERAP1, we sequenced the peptide products of ERAP1 incubation with the long peptides in **Figure 3** by Mass Spectrometry (**Figure 4A** **)**. The results demonstrated that majority of the peptides recovered from ERAP1-ALKRQGRTLYGF digestions were the octamer peptide QGRTLYGF (60% fraction), and only 10% of peptides remained longer than nonamers. In contrast, ERAP1 trimming of the peptide LARRYGDVFQI yielded mainly nonamers, while digestions of peptide SLARYGKSPYLY yielded an abundance of octamers (60%) in addition to a large remaining fraction of the untrimmed full-length peptide (40%). Concordantly, ERAP2 activity remained partially inhibited even by a decreased pool of long peptides. Finally, for the peptide QLRRFGDKLNF, ERAP1 failed to produce a significant pool of octameric products, and so the resulting pool of peptides were mainly composed of nonomers or longer peptides that were resistant to ERAP2 digestion. Taken together, these observations demonstrate that ERAP1 can rescue ERAP2 activity if the long peptide inhibitor is a good substrate for ERAP1. Additionally, we observed that the presence of octamer RYQKSTEL and hexamer QKSTEL in the assay did not affect ERAP2 enzymatic activity whereas the long peptide EIRRYQKSTEL inhibited ERAP2 (**Figure 4B**). While these results do not agree with the reported observations using SIINFEHL peptide (15), they can simply be reflecting differences in the internal sequence of the peptide and especially in the P1 residues (24, 25).

## Discussion

The peptidase activities of ERAP1 and ERAP2 are clearly intertwined and complementary. Here, we demonstrate that the preferred ligands for each enzyme are different, and that the products of the enzymatic digestion by one enzyme can be inhibitory or stimulatory for the other. This mechanistic insight shines new lights on how these 2 aminopeptidases work in concert to optimally promote the generation of the peptidomes presented on cell surface (5).

Specifically, we demonstrated that octameric peptides, which can be generated by trimming of the longer precursors, will subsequently suppress ERAP1 trimming activity of the long peptide substrates. Interestingly, this effect was quite specific to octamers and longer peptides, as heptameric peptides instead either had no effect on ERAP1 activity or in some occasions even enhanced ERAP1 activity as it was previously reported for hexamers (16). One can explain these observations by imagining that short octameric peptides can occupy the ERAP1 substrate pocket acting essentially as a competitive inhibitor by lowering the pool of available ERAP1 binding sites for the other peptides. However, suppression of ERAP1 activity in this manner is a highly dynamic process. If additional longer cytosolic peptides are transported into the ER, or octamers are removed from the ER peptide pool, then the full ERAP1 trimming activity is restored. One clear mechanism for depleting the available octameric peptide pool is through the destruction by ERAP2. Given that ERAP2 already resides in the ER along with ERAP1, this mechanism might restore ERAP1 activity more efficiently than that of the rate of peptides transported into the ER.

We also found that ERAP2 activity was inhibited by the presence of long peptide substrates. Importantly, ERAP1 activity can decrease the pool of long peptides and generate shortened versions optimal for ERAP2. There was not, however, a one-size-fits-all effect between the pool of peptide substrates and the activities of ERAP1 and ERAP2. In fact, we observed 3 potential unique outcomes from the addition of peptides pretreated with ERAP1 on the activity of ERAP2. When ERAP1 could generate a preponderance of octamers and nonamers from a longer peptide substrate, essentially a total recovery in ERAP2 activity was observed. If ERAP1 generated a smaller fraction of shorter peptides, then there was a partial recovery in ERAP2 activity. Finally, if ERAP1 failed to sufficiently trim a long peptide inhibitor to nonamers or octamers, ERAP2 activity was ablated due to the predominance of long peptides. The different outcomes observed is likely due to different activities of ERAP1 against the peptides of different sequence. We observed that peptides whose P1 residues are good substrates for ERAP1, led to better recovery of ERAP2 activity.

One limitation of the current study is that we focused on the most common ERAP1 haplotype (Hap2), which carries the variant K528. This enzymatic isoform is known to be highly active. It is possible that other ERAP1 haplotypes with different amino acid variants and differing activity have slightly different collaborative behaviors with ERAP2. However, since the main amino acid change in the other haplotypes is thought to primarily alter the kinetics of ERAP1 domain rearrangements during enzymatic trimming (12), these results can likely be safely extrapolated to other ERAP1 variants.

### Proposed model of ERAP1 and ERAP2 synergy: Cross modulation

**Figure 5:**
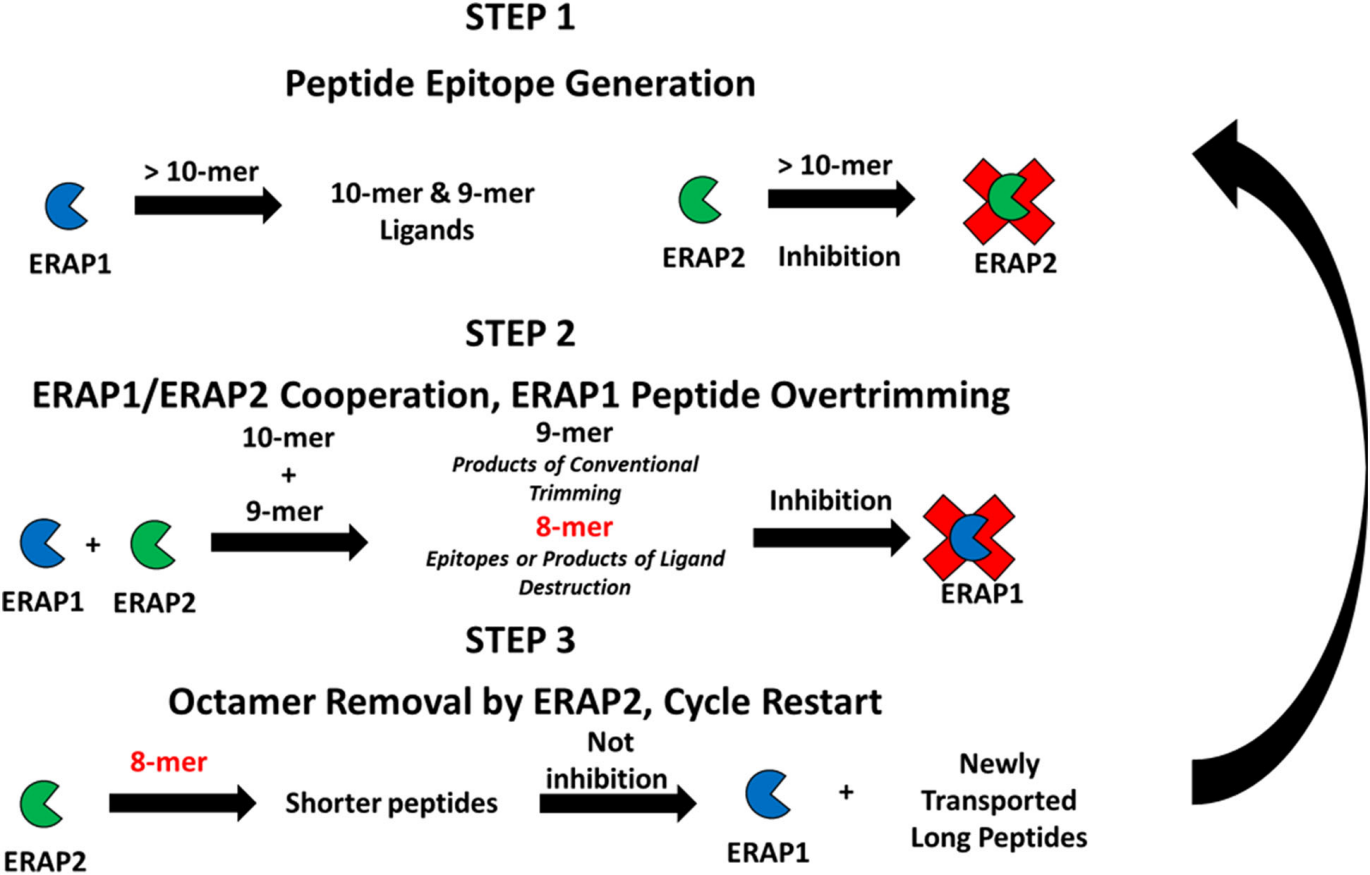
Proposed cross-feedback modulation model of ERAP1 and ERAP2 peptide trimming ERAP1 trims long peptides transported into the ER, long peptides may bind unproductively to inhibit ERAP2 activity against newly generated shorter peptide substrates. ERAP1 and ERAP2 cooperate to produce peptides of ideal length for antigen processing. ERAP2 can rescue ERAP1 octamer-dependent inhibition resulting from the overtrimming of epitopes by the combined activities of ERAP1 and ERAP2.

To clarify how these interactions of each enzyme with different ligands and their products could affect the process of peptidome trimming, we propose the following unifying model for antigen generation, following a peptide through its steps from import into the ER to availability for binding to MHC molecules (**Figure 5**).

Step 1 Long peptide import – ERAP1 activity predominates: A high proportion of peptides translocated into the ER are longer than 9 amino acids that are optimal length for ERAP1 activity. Also, a portion of the long peptide can bind ERAP2 peptide binding groove and inhibit its activity. ERAP1 trims these longer peptides until their length is optimal for class I antigen presentation (generally 8-11 amino acids long, dependent on HLA allele).

Step 2 Cooperative trimming of intermediate length peptides – balance of ERAP1 and ERAP2 activity: For peptides of intermediate length, nonomers and decamers, in the ER pool, trimming by both ERAP1 and ERAP2 can occur. Based on the sequence preference of the individual enzymes, the cooperative activity of these two enzymes enables generation of a variety of peptides for binding to MHC-I. Decamers with basic residues in position P1 are poorly trimmed by ERAP1, but they can instead be trimmed by ERAP2. In that scenario ERAP2 can help ERAP1 generate appropriately sized peptides of favored compositions to bind the MHC-I peptide groove. Conversely, ERAP2 can bind nonamers with highly resistant P1 residues, therefore placing ERAP2 as a protector of rare peptides by sequestering them from over-trimming by ERAP1. Simultaneously, the combined ERAP1 and ERAP2 trimming activities in the ER can reduce concentration of the optimal nonamer ligands, or produce octamers that could form shorter epitopes.

Step 3 Short peptide over-trimming and elimination – ERAP2 activity predominates: For octamers that reach a sufficient ER concentration, ERAP1 activity is suppressed. Instead, ERAP2 activity is favored when high amounts of octamers are present, and the octamer pool gradually decreases. Because ERAP2 affinity and activity is higher against octamers, destruction of nonamer epitopes is suppressed. Eventually enough new peptide precursors are translocated into the ER and the ratios of longer peptides to octamers increases. A significant reduction of octamers by ERAP2 and the return to a peptide pool composed of longer peptides reactivates ERAP1 activity; ERAP2 is slowly inhibited and the state of ER aminopeptidase activity returns to step 1; the cycle restarts.

The above proposed cycle is of course a simplified version of the complex interplay between peptides of different lengths and ERAP1 and ERAP2, with changes in the ER peptide pool likely happening with rapid dynamics. By synergizing and cross modulating each other’s activities, proper feedback between ERAP1 and ERAP2 activities will have important effects in optimizing the peptidome presented by HLA molecules. Clearly, perturbations of this finely regulated pathway, such as in virally infected or transformed cells, can affect immunosurveillance by cytotoxic T cells and even NK responses (26–31). Furthermore, understanding how this cross-modulatory behavior is affected by different haplotypes of ERAP1 or alternative isoforms of ERAP2 whose function has not been yet characterized (32), will be a fruitful area of future investigation. The critical role of the ERAP1 and ERAP2 aminopeptidases in antigen processing presents a unique target to affect T cells responses with relevance to infection, autoimmunity, and immunosurveillance of cancer.

## Data availability statement

The raw data supporting the conclusions of this article will be made available by the authors, without undue reservation.

## Author contributions

AM-E designed, performed, analyzed and wrote the paper. JP performed experiments and wrote the paper. JR performed experiments, JL, NS and SS-N supervised and provided funding. SS-N also wrote the paper. All authors contributed to the article and approved the submitted version.

## Supplementary material

Supplementary Figure S1 Trimming of short peptides by ERAP1: A 9-mer peptide RRYQKSTEL was incubated with ERAP1 at a E:S ratio of 1:10 (w/w) at various times from 5 min to overnight. Yields are relative to the total amount of peptide estimated as the added intensity of ion peaks corresponding to each peptide species by MALDI-TOF MS spectrometry of final reaction products. In Table B the % of each species at each time point during the course of experiment are shown.

Supplementary Figure S2 Trimming of short peptides by ERAP2: Panel A, 8-mer peptide RYQKSTEL was incubated with ERAP2 at a E:S ratio of 1:10 (w/w) for 5 minutes and the ion peaks corresponding to each peptide species were identified by MALDI-TOF MS spectrometry. Panel B. 11-mer Peptide LARRYGDVFQI, was incubated with ERAP1 (top panel), with ERAP1 and the presence of an 8-mer (lower left) and ERAP1 and ERAP2 with an 8mer (lower right) for 16 minutes.

Supplementary Figure S3 Rescue of ERAP1 inhibition by ERAP2 activity due to trimming of octamer. The indicated peptides were incubated at a ratio of E:S of 1:10 (w/w) with ERAP1 alone (black), or coincubation of ERAP1 only with 100 μM RYQKSTEL (white) and of ERAP1 plus ERAP2 with 100 μM RYQKSTEL (grey). Yields relative to the total amount of the peptides were estimated as the total intensity of the ion peaks corresponding to each peptide species by MALDI-TOF MS spectra. The data represent means and SD of 3 independent experiments.

Supplementary Figure S4 ERAP2 does not enhance ERAP1 trimming of long peptides or epitope generation, but enhances epitope destruction. Peptides were incubated with ERAP1 (blue) and ERAP1 plus ERAP2 (red) at a E:S ratio of 1:10 (w/w) for 32 min. The effect of ERAP1 plus ERAP2 activity for each peptide condition was normalized to that of ERAP1 alone and presented as percent activity.

Supplementary Figure S5 Trimming Substrate Preferences for ERAP1 & ERAP2. A X-GRFSGLLGR peptide series were subjected to ERAP1 & ERAP2 digestion for 15 min at the ratio of 1:10 (w/w), where X represents one of 20 possible N-terminal amino acids.
