# Supplementary figures and images for: The ER Aminopeptidases, ERAP1 and ERAP2, synergize to self-modulate their respective activities
Source: Front Immunol. 2022 Dec 8;13:1066483. doi: 10.3389/fimmu.2022.1066483 (PMC9774488; doi:10.3389/fimmu.2022.1066483)

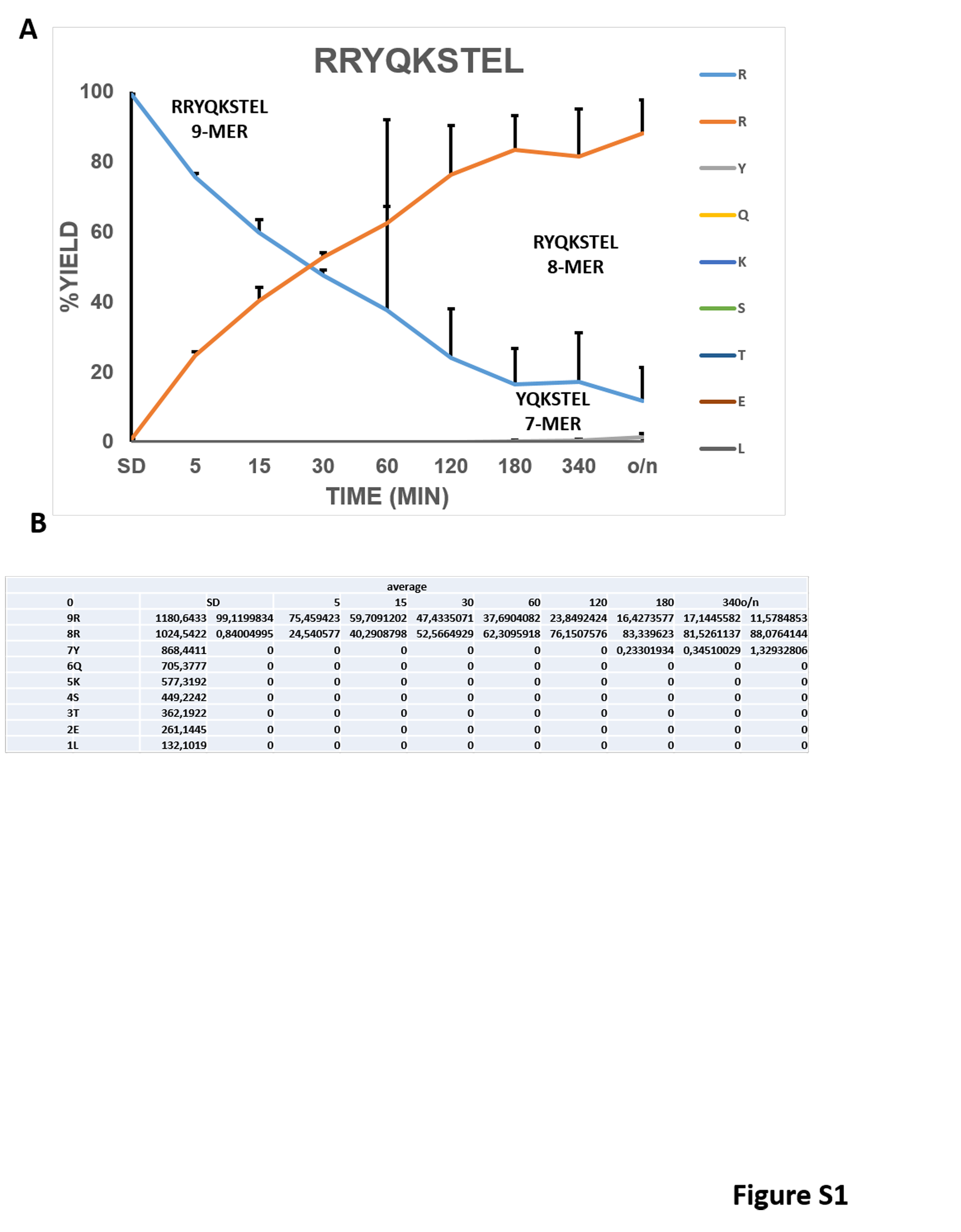

Supplement: Supplementary Figure S1 — Trimming of short peptides by ERAP1: A 9-mer peptide RRYQKSTEL was incubated with ERAP1 at a E:S ratio of 1:10 (w/w) at various times from 5 min to overnight. Yields are relative to the total amount of peptide estimated as the added intensity of ion peaks corresponding to each peptide species by MALDI-TOF MS spectrometry of final reaction products. In Table B the % of each species at each time point during the course of experiment are shown. [file DataSheet_1.zip › Supplementary Material/Figure S1.tif]

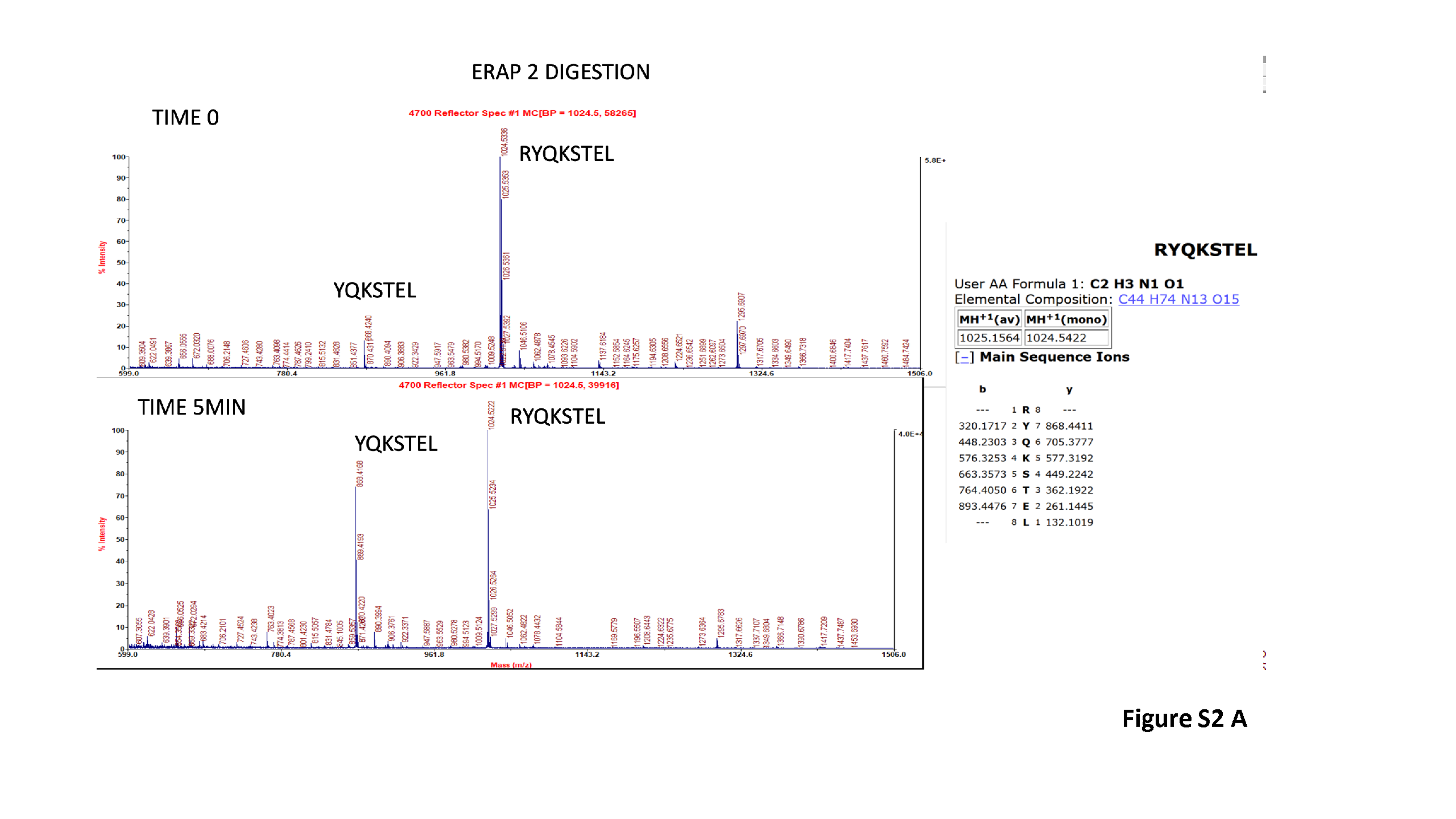

Supplement: Supplementary Figure S1 — Trimming of short peptides by ERAP1: A 9-mer peptide RRYQKSTEL was incubated with ERAP1 at a E:S ratio of 1:10 (w/w) at various times from 5 min to overnight. Yields are relative to the total amount of peptide estimated as the added intensity of ion peaks corresponding to each peptide species by MALDI-TOF MS spectrometry of final reaction products. In Table B the % of each species at each time point during the course of experiment are shown. [file DataSheet_1.zip › Supplementary Material/Figure S2A.tif]

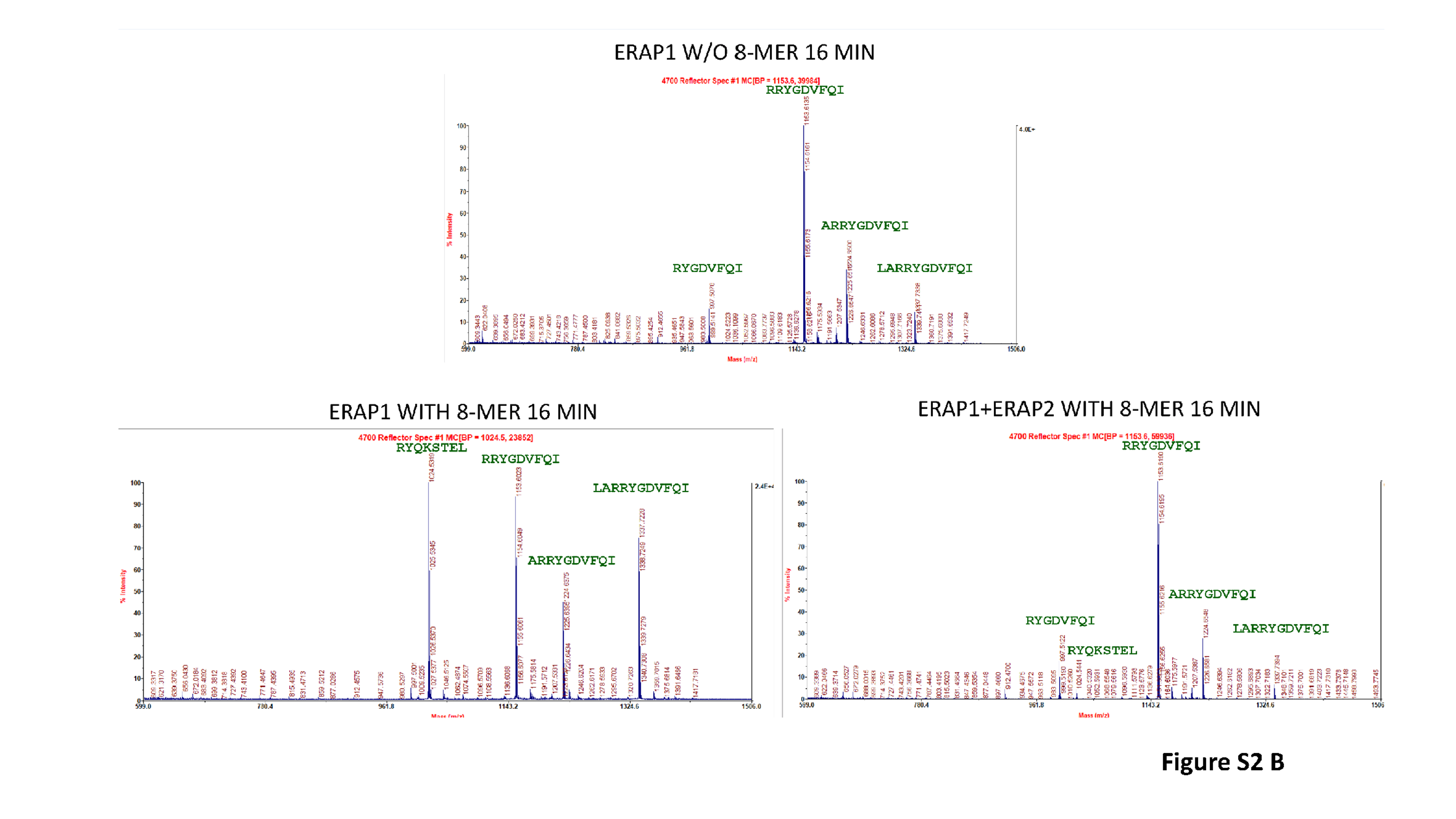

Supplement: Supplementary Figure S1 — Trimming of short peptides by ERAP1: A 9-mer peptide RRYQKSTEL was incubated with ERAP1 at a E:S ratio of 1:10 (w/w) at various times from 5 min to overnight. Yields are relative to the total amount of peptide estimated as the added intensity of ion peaks corresponding to each peptide species by MALDI-TOF MS spectrometry of final reaction products. In Table B the % of each species at each time point during the course of experiment are shown. [file DataSheet_1.zip › Supplementary Material/Figure S2B.tif]

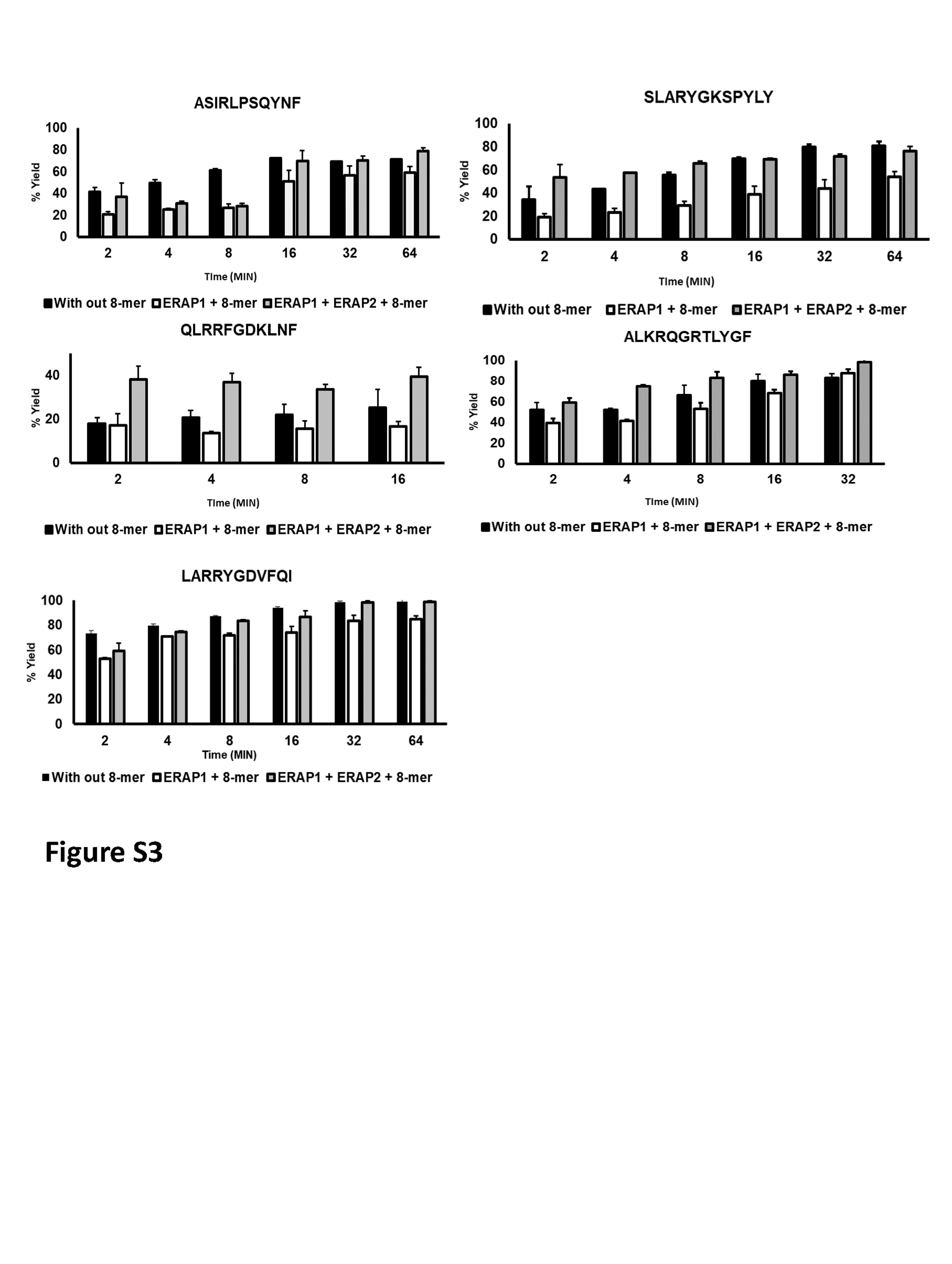

Supplement: Supplementary Figure S1 — Trimming of short peptides by ERAP1: A 9-mer peptide RRYQKSTEL was incubated with ERAP1 at a E:S ratio of 1:10 (w/w) at various times from 5 min to overnight. Yields are relative to the total amount of peptide estimated as the added intensity of ion peaks corresponding to each peptide species by MALDI-TOF MS spectrometry of final reaction products. In Table B the % of each species at each time point during the course of experiment are shown. [file DataSheet_1.zip › Supplementary Material/Figure S3.tif]

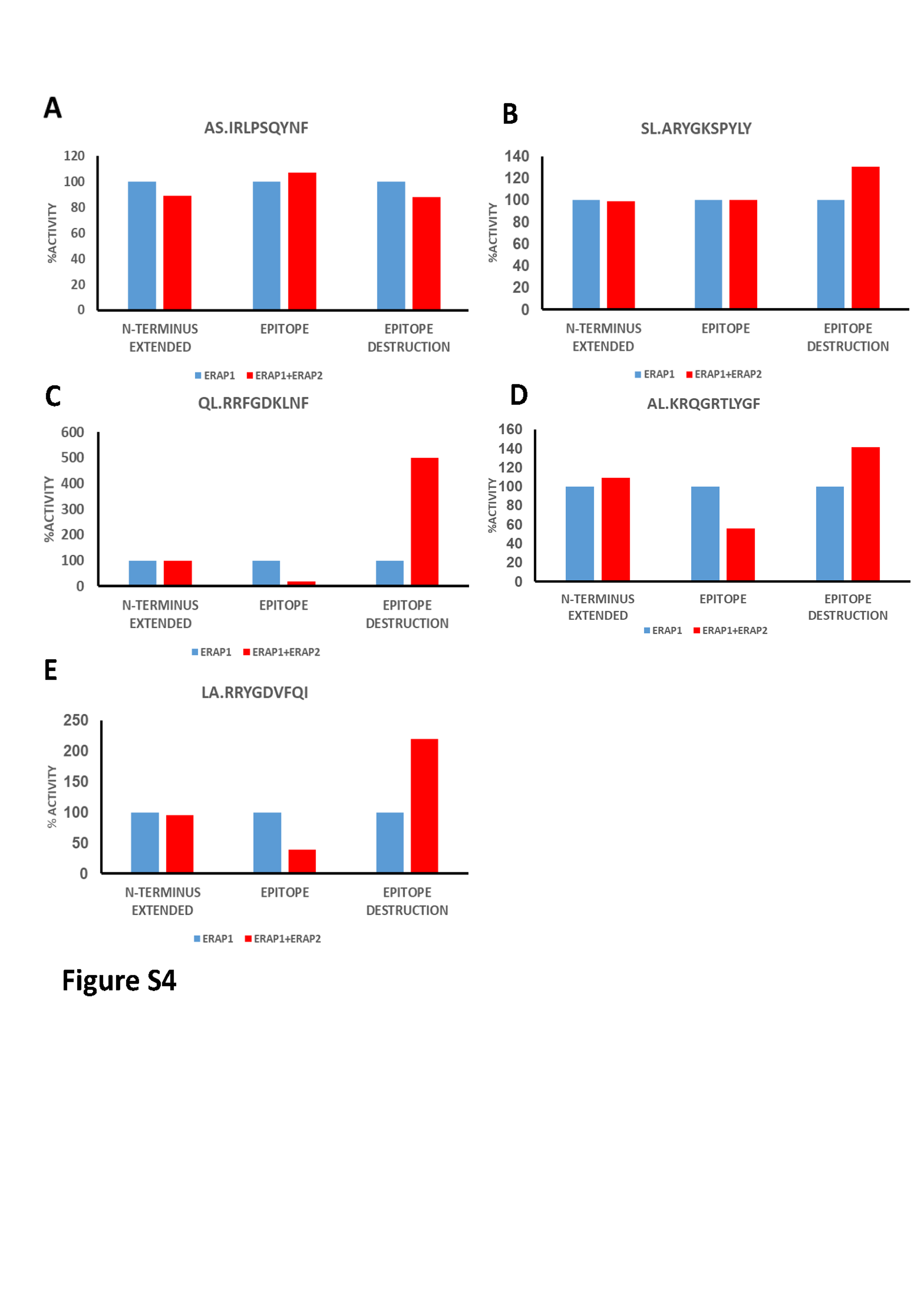

Supplement: Supplementary Figure S1 — Trimming of short peptides by ERAP1: A 9-mer peptide RRYQKSTEL was incubated with ERAP1 at a E:S ratio of 1:10 (w/w) at various times from 5 min to overnight. Yields are relative to the total amount of peptide estimated as the added intensity of ion peaks corresponding to each peptide species by MALDI-TOF MS spectrometry of final reaction products. In Table B the % of each species at each time point during the course of experiment are shown. [file DataSheet_1.zip › Supplementary Material/Figure S4.tif]

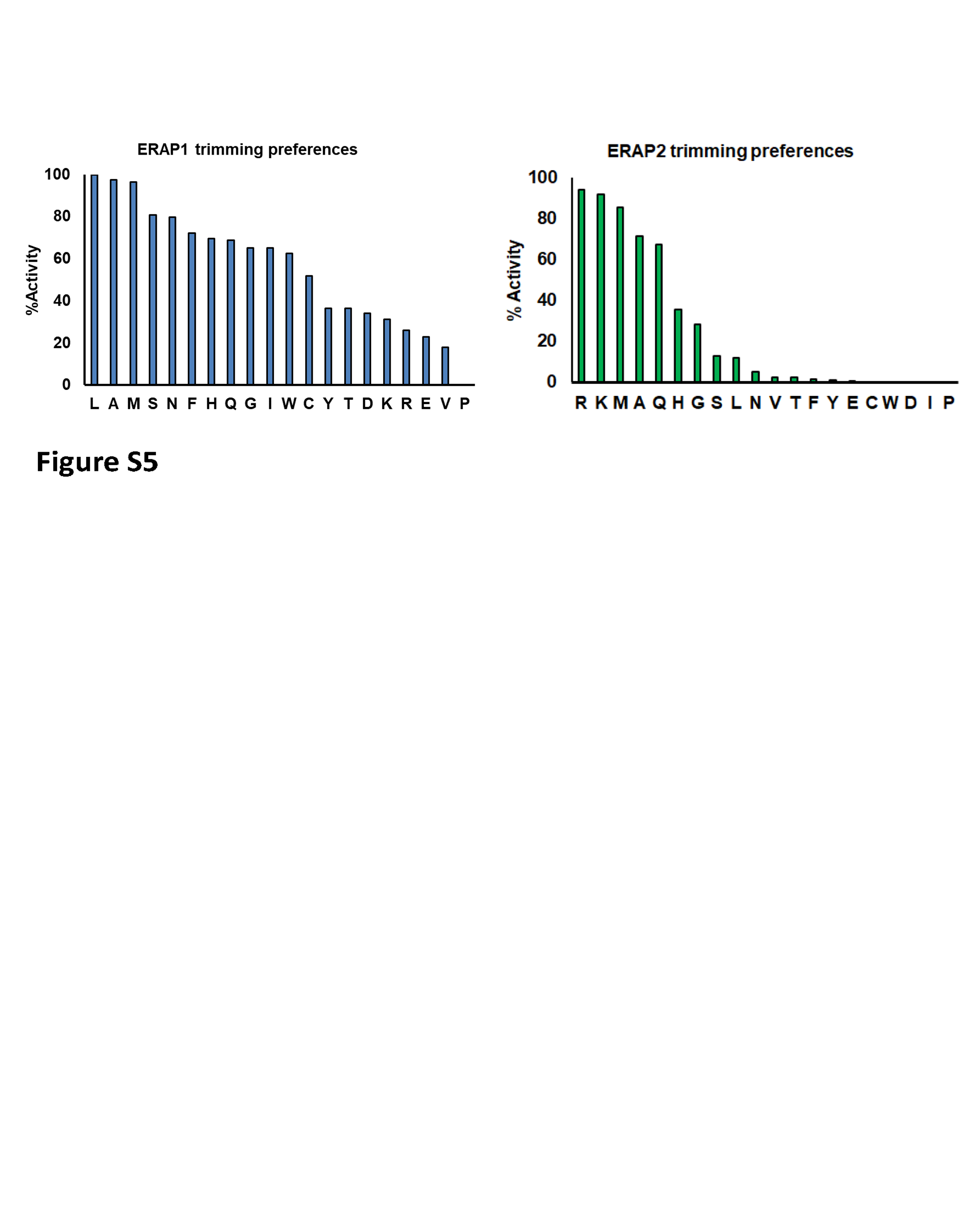

Supplement: Supplementary Figure S1 — Trimming of short peptides by ERAP1: A 9-mer peptide RRYQKSTEL was incubated with ERAP1 at a E:S ratio of 1:10 (w/w) at various times from 5 min to overnight. Yields are relative to the total amount of peptide estimated as the added intensity of ion peaks corresponding to each peptide species by MALDI-TOF MS spectrometry of final reaction products. In Table B the % of each species at each time point during the course of experiment are shown. [file DataSheet_1.zip › Supplementary Material/Figure S5.tif]
